# Value of Probiotics on Outcome in Patients Following Liver Surgery: A Systematic Review and Meta-Analysis

**DOI:** 10.3390/medicina61061068

**Published:** 2025-06-10

**Authors:** Robert Karitnig, Andreas Bogner, Nora Jahn, Christos Vlachos, Andri Lederer, Antonia Geisler, Robert Sucher, Hans Michael Hau

**Affiliations:** 1 Department of General, Visceral and Transplant Surgery, Medical University of Graz, 8010 Graz, Austria; robert.karitnig@medunigraz.at (R.K.); robert.sucher@medunigraz.at (R.S.); hans.hau@medunigraz.at (H.M.H.); 2 Department for Anesthesiology and Intensive Care Medicine, Medical University of Graz, 8010 Graz, Austria

**Keywords:** probiotics, synbiotics, prebiotics, liver surgery, liver transplantation, liver resection, hepatectomy, prognosis, meta-analysis, randomized controlled trial

## Abstract

*Background and Objectives:* The gut–liver axis plays a crucial role in the development of post-surgical infections. Surgery-induced dysbiosis can lead to increased bacterial translocation, impairing the liver’s detoxification capacity and negatively affecting surgical outcomes. Following liver surgery, approximately a third of the patients develop bacterial infections, with a high risk of bacteremia or even sepsis-related liver failure and death. The potential advantages of administering pro- or synbiotics before/after surgery remain a topic of discussion. Therefore, a systematic review of randomized clinical trials comparing patients with and without supplementation and their outcomes and effects after liver resection (LR) or liver transplantation (LT) was conducted. *Materials and Methods:* A computer-based search of electronic databases was conducted to gather randomized controlled trials (RCTs) that focused on probiotic/synbiotic use during the perioperative period for liver surgery patients. Two researchers independently screened the studies, extracted the data, evaluated the risk of bias, and performed a meta-analysis using RevMan Web. *Results:* Our research revealed 19 relevant randomized controlled studies that included a total of 1698 patients on the perioperative use of pro-/symbiotic administration in liver surgery. Eight studies were performed on liver transplantation (LT), and 11 studies were performed for liver resection (LR). The results of the meta-analysis demonstrated that the probiotic group exhibited lower rates of postoperative infectious complications (OR = 0.34; 95%CI 0.25 to 0.45; *p* < 0.0001), hospital stay duration (SMD = −0.13; 95%CI −0.25 to −0.00; *p* = 0.05), lower serum endotoxin levels (SMD = −0.39%CI −0.59 to −19; *p* < 0.0001), and white blood cell counts (SMD = −SMD = −0.35; 95%CI −0.56 to −0.13; *p* = 0.002) compared to the control group. Further, with regard to liver function, we observed significant postoperative differences in alanine aminotransferase (ALT)-levels (SMD = −0.46; 95%CI −0.63 to −0.29; *p* < 0.0001), aspartate aminotransferase (AST) levels (SMD = −0.53; 95%CI −0.71 to −0.34; *p* < 0.0001), bilirubin levels (SMD = −0.35; 95%CI −0.50 to −0.19; *p* < 0.0001), and international ratio (INR) levels (SMD = −0.1; 95%CI −0.12 to −0.08; *p* ≤ 0.0001), favoring the symbiotic group compared to the control group. *Conclusions:* The use of pro-/synbiotics during the perioperative period reduces the risk of postoperative infections, support postoperative liver function, and recovery and shortens hospital stays for liver surgery patients. However, they do not appear to particularly aid in inflammation reduction.

## 1. Introduction

Liver cirrhosis and hepatic tumors are frequently the end stages of chronic liver disease. Surgical treatment, including liver resection (LR) or liver transplantation (LT), is typically considered the preferred approach in most situations [1].

LR, which is primarily performed for tumors, carries a mortality rate of 3.5% and a morbidity rate ranging from 10 to 15% [2,3]. However, the high rate of postoperative complications significantly impacts patient outcomes [4,5]. Statistics show that approximately 30% of patients who undergo liver resection experience postoperative infections, with around 10% developing intra-abdominal sepsis [2,6,7]. For patients undergoing extensive liver resection, the rate of postoperative infections can rise to 34% [8]. The onset of postoperative bacterial infections not only raises the risk of liver failure by about 50%, but it also increases the mortality rate by over 40% [2,6,9]. General risk factors include malnutrition, surgical trauma, and parenteral nutrition. The trauma caused by liver surgery can impair intestinal barrier function, leading to an imbalance in gut microbiota [10]. Furthermore, the use of antibiotics, analgesics, and proton pump inhibitors during the perioperative period can worsen this imbalance, increasing the likelihood of infections [2,11,12,13]. Postoperative infections are not only a critical factor affecting patient prognosis, but they also significantly raise the economic burden on patients. Preoperative malnutrition significantly raises the risk of infectious complications following LT, while impaired liver function prior to LT may be linked to postoperative bacteremia [14,15]. Additionally, immunosuppression after LT further amplifies the risk of infections. Stress-induced dysbiosis contributes to bacterial translocation, increasing vulnerability to infections. Despite advances in antibiotic treatment and infection control measures, the incidence of sepsis continues to rise [16]. The growing global concern over antimicrobial resistance underscores the need for new strategies to mitigate infection risks in surgical patients; thus, reducing the incidence of postoperative infection is an urgent clinical challenge. Probiotics, pre-biotics, and synbiotics—as nutritional supplements—have been widely used in the management of respiratory and gastrointestinal infections, acute diarrhea, and antimicrobial-associated diarrhea [2,17,18,19,20].

They are also considered promising treatments for preventing postoperative infections in the gastrointestinal and hepato–pancreatic–biliary systems [2,21,22]. Their mechanisms of action include the competitive exclusion of harmful pathogens, direct antibacterial effects, modulation of intestinal mucosal pH, and prevention of bacterial translocation by promoting tight junctions in intestinal epithelial cells [2,23,24]. Oral probiotics can improve gut flora diversity and stimulate the production of anti-inflammatory cytokines [24].

However, the effectiveness of probiotics in patients undergoing liver surgery has not been thoroughly fully investigated. Existing guidelines for enhanced perioperative recovery, including those from the European Association for the Study of the Liver and the American Association for the Study of Liver Diseases, do not include probiotics in post-hepatectomy care, since their benefit is still under debate [25].

Two prior meta-analyses to this topic show that perioperative probiotic administration could significantly reduce the incidence of infectious complications after hepatectomy [2,26]. However, both these meta-analyses included many retrospective studies and did not address important aspects after liver surgery, such as inflammatory indices and liver function, in its evaluations, thus limiting the generalizability of its findings. Additionally, recent new and promising studies on the topic have been published, necessitating an updated meta-analysis [17,27,28,29,30,31]. Consequently, this study conducted a systematic review and meta-analysis of only randomized controlled trials (RCTs) to assess the effects of pro-/synbiotics on the prognosis and outcome of patients undergoing liver surgery. The main focus was on evaluating the impact of probiotics on postoperative liver function, inflammatory markers, infectious complications, and recovery. The goal was to provide a scientific basis and reference for the clinical use of probiotics.

## 2. Methods

### 2.1. Literature Retrieval Strategy

This meta-analysis was performed in accordance with the Preferred Reporting Items for Systematic Reviews and Meta-Analyses (PRISMA) guideline. The study protocol was registered in a prospective registry of systematic reviews (CRD number: 1022218). The comprehensive systemic search and collection of randomized controlled trials (RCTs) evaluating the effectiveness of perioperative oral probiotics in patients undergoing liver surgery were conducted through detailed searches of several databases. These included PubMed, Cochrane Library, Web of Science, Embase, Central, and the China BioMedical Literature Database (CBM). The search covered the period from the inception of each database to 30 January 2025. In addition, the reference lists of the identified studies were manually reviewed to ensure that all the relevant literature was included. The search strategy used a combination of free-text terms and subject headings, with the following key terms: “probiotic, probiotics, synbiotics, symbiotic, prebiotic, prebiotics, microecological regulators, microecological agents, liver surgery, hepatectomy, liver resection, and liver transplantation”.

### 2.2. Inclusion and Exclusion Criteria

▪ Inclusion Criteria:
  - **Population (P)**: Adults aged 18 or older who required liver segmentectomy, liver lobe resection, hemihepatectomy, or liver transplantation.
  - **Intervention (I)**: The experimental group received oral microecological agents (probiotics, prebiotics, synbiotics) with no restrictions on the type or dosage of agents. The control group received either a placebo or no treatment.
  - **Comparison (I)**: Comparison between the probiotic group and the control group to assess the impact of probiotics on the postoperative prognosis of hepatectomy patients.
○ Outcome (O):
  **1.**  **Infection-related indicators**: white blood cell count (WBC), serum levels of endotoxin, C-reactive protein (CRP), procalcitonin (PCT), and interleukin-6 (IL-6);
  **2.**  **Perioperative liver function and recovery indices**: alanine aminotransferase (ALT), aspartate aminotransferase (AST), bilirubin levels, and international normalized ratio (INR);
  **3.**  **Postoperative outcome**: Postoperative infection rate, length of hospital stay.
○ Study Design (S): Only RCTs were included.
▪ Exclusion Criteria:
  - Studies published in other languages than English/no English translation version of the study;
  - Data that could not be converted to meet the study requirements;
  - Inability to obtain the full text of a study;
  - Conference abstracts, editorial articles, case reports, non-randomized controlled studies, studies including children or animals, in vitro studies and systematic reviews and meta-analyses were excluded.

### 2.3. Data Extraction

Two researchers (H.M.H., R.K.) independently reviewed the literature, and data extraction was performed with cross-verification. Any disagreements were resolved through discussion, and a third researcher (A.B.) was consulted if necessary. The literature selection process involved initially reviewing the title and abstract, followed by a full-text reading to confirm inclusion, with exclusion of the obviously irrelevant studies. The extracted data included title, country, publication date, first author, type of surgery (liver resection, liver transplantation), number of study participants per group, intervention details for both groups, time and duration of microecological agents’ treatment, study type, and outcome indicators.

### 2.4. Quality Assessment and Risk of Bias Assessment

The risk of bias for each included study was assessed independently by two investigators using the RCT risk of bias assessment tool from the Cochrane Manual [32] and Jadad Score [33] which was ultimately not included in Table 1 due to issues related to the handling of the measurement. In case of discrepancies, they resolved issues through discussion, or by involving a third investigator. The assessment focused on the adequacy of random allocation, allocation concealment, methodological accuracy, selective reporting of outcomes, completeness of data, and blinding of participants, treatment protocols, and study results. Each element was categorized as “high risk of bias”, “low risk of bias”, or “unclear risk of bias”.

### 2.5. Statistical Analysis

Statistical analysis was performed using RevMan online. For continuous data, mean differences (MD) or standardized mean differences (SMD) were used as effect size measures, while odds ratios (OR) were applied to categorical data. Each effect size was accompanied by its point estimate and 95% confidence intervals (CI). Heterogeneity among the included studies was assessed using the Q test, combined with the I^2^ value to measure the degree of heterogeneity. If statistical heterogeneity was low (*p* > 0.1 and I^2^ < 50%), then a fixed-effect model was used for meta-analysis. If heterogeneity was high (*p* < 0.1 and I^2^ ≥ 50%), then a random-effects model was applied. Sensitivity analysis—including only randomized controlled studies—was conducted by excluding one study at a time to identify potential sources of heterogeneity.

**Table 1:** Baseline characteristics of included studies.

Study Characteristics				Number of Cases			Treatment Protocol				Patient Characteristics			
Year	Author	Country	Period	Total	Contro	Probiotics	Pro/Synbiotic	Control Group	Doze	Treatment Duration/Postoperativ Start	Age	SD	Gender/Control (m/f)	Kind of Surgery
2002	Rayes [34]	Germany	1997–1999	63	32	31	*Lactobazillus casei*	placebo	n.m.	12 after surgery/first POD	50.3	2.2	15/17	LTX
2005	Rayes [35]	Germany	2003–2004	66	33	33	*Pediacoccus pentosaceus*, *Leuconostoc mesenteroides*, *Lactobacillus paracasei* ssp. *Paracasei*, *L. plantarum*	low fibre formula	1 dose/2× daily	14 after surgery/first POD	51.5	2.5	16/17	LTX
2005	Kanazawa [36]	Japan	2000–2002	44	23	21	Yakult BL Seichoyaku	placebo	3 g/d	POD 1-14/first POD	63.7	9.6	14/9	LR
2006	Sugawara [37]	Japan	2003–2005	81	40	41	*Lactobacillus casei*, *Bifidobacterium breve*	placebo	15 g/day	14 before and after surgery/first POD	63.1	8.8	23/18	LR
2010	Rifatbegovic [38]	France	2006–2008	120	60	60	synbiotic composition	placebo	n.m.	3 days before and 7 days after/first POD				LR
2011	Usami [10]	Japan	2005–2008	61	29	32	Yakult BL Seichoyaku	no synbiotics	n.m.	14d before and 14 d after/first POD	65.4	9.8	16/3	LR
2011	Eguchi [39]	Japan	2005–2009	50	25	25	Yakult BL antiflatulent	placebo	15 g/d	14d before and 14 d after/first POD	56.5	9.5	13/12	LTX
2012	Rayes [40]	Germany	2007–2008	19	10	9	*Pediococcus pentosaceus*, *Leuconostoc mesenteroides*, *Lactobacillus paracasei* ssp. *Paracasei*, *Lactobacillus plantarum*	fibres	1 dose/2× daily	before and 10 days after/first POD	60.1	13.5	6/4	LR
2013	Zhang [41]	China	2011	67	33	34	synbiotic composition	low fibre	n.m.	7 days PO/first POD	56	11	17/16	LTX
2014	Russolillo [42]	Italy	2008–2010	40	20	20	Prebiotic (Medibase)	no synbiotics	4.5 g	1 week before until discharge/as tolerated	63.5	12.4	10/10	PPPD, TP, MajHep
2015	Liu [43]	China	2007–2013	134	68	68	synbiotic composition	maltodextrin	n.m.	6 d pre and 10 post/first POD	62.8	17.4	35/33	LR
2017	Grat [44]	Poland	2012–2015	44	23	21	Lactococcus lactis PB411, Lactobacillus casei PB121, Lactobacillus acidophilus, Bifidobacterium bifidum PB211	placebo	1 capsula/daily	time of listing until surgery/not postoperativ	50.9	5.1	20/6	LTX
2020	Iida [45]	Japan	2011–2017	284	60	60	*Clostridium butyricum* + *Prebioticum*	none	6 g/d + 12 g/d	2 weeks before and after/first POD	66.3	13.6	50/10	LR
2022	Huang [17]	China	2018–2020	100	50	50	*Clostridium butyricum*	placebo	3 × 2 Tablets/daily	3 days before and 4 after/first POD	50.3	8.3	44/6	LR
2022	Mallick [27]	India	2016–2017	100	50	50	Prowel (Prepro arm)	placebo	1 capsula/dail	2 d before and 14 d after/first POD	49.9	6.9	45/5	LDLT recipient
2022	Roussel [28]	France	2013–2018	54	27	27	Lactibiane Tolerance	placebo	2×/daily	14 days before surgery/not postoperativ	66.5	7.7	23/4	LR
2022	Wu [29]	China	2018–2020	110	55	55	*Clostridium butyricum*	placebo	2 capsuls/3× per day	3 d pre and 4 d post/first POD	55.6	15.5	37/18	LR
2023	Ramachandran [31]	India	2021–2022	215	107	108	synbiotic composition	placebo	2/daily for 6 weeks	for 6 Weeks/n.m.	48	IQR: 24–77	94/15	LTX
2024	Yoshiya [30]	Japan	2018–2023	211	176	34	*Lacticaseibacillus paracasei*, *Bifidobacterium breve*	no synbiotics	3×/d	5 d before and 5 after/first POD	56.6	10.60	79/97	LDLT donor

SD—standard deviation; POD—postoperative day; LTX—liver transplantation; LR—liver resection; m—male; f—female.

## 3. Results

### 3.1. Literature Search Outcomes

**Figure 1:**
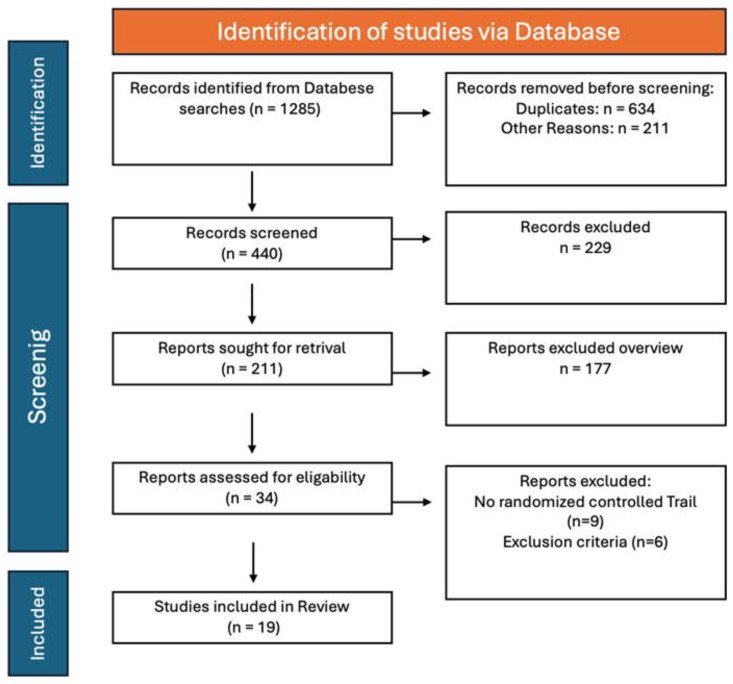
Flow chart of the literature selection.

Following the initial search, 1285 relevant articles were initially identified. After the first round of screening, 634 articles were excluded due to duplicates. Another 211 articles were ruled out based on their titles and abstracts as they did not align with the research topic. Further review of the full texts led to the exclusion of an additional 440 articles that did not meet the inclusion criteria. In the end, 19 randomized controlled trials (RCTs) [10,17,27,28,29,30,31,34,35,36,37,38,39,40,41,42,43,44,45] were selected for the meta-analysis. A flowchart of the retrieval process is presented in Figure 1.

### 3.2. Study Characteristics

Of these 19 included studies, all were published in the English language with publication dates ranging from 2002 to 2024. Eight studies were performed on liver transplantation (LT), and 11 studies were performed on liver resection (LR). The studies involved sample sizes from 19 to 215 participants, totaling 1698 patients who underwent liver surgery. Among them, 777 were in the probiotic/synbiotic intervention group, and 921 were in the control group. The key characteristics of these studies are summarized in Table 1. A total of nine different pro-/synbiotics were used in these studies. Study sites were in Japan (*n* = 6), Germany (*n* = 3), Poland, Bosnia, France, India (*n* = 2), Italy, and China (*n* = 4). The mean duration of pro-/synbiotic administration was 17.23 ± 9.98 days. In nine studies, probiotics were used, while synbiotics were administered in 10 trials. The most commonly utilized comparators were placebo/other (*n* = 14) and no intervention (*n* = 5). In the case of postoperative administration, which applied to 16 studies, probiotic therapy was initiated within the first 24 h, either orally or via an existing enteral feeding tube. In the other studies, administration was either only preoperative (*n* = 2) or with an unclear start time postoperatively (*n* = 1), as the studies did not provide further details.

The contraindications for the administration and use of probiotics in the included studies were as follows: Kidney diseases and renal insufficiency, postoperative interventions involving changes in gastrointestinal anatomy such as bilio-digestive anastomoses or Roux-en-Y reconstructions, cerebral diseases and impairments, liver diseases such as cirrhosis, immune system impairments like immunodeficiency syndromes, patient compliance, and emergency surgeries were the most common contraindications and exclusion criteria for the administration of probiotics.

### 3.3. Bias Risk Assessment in Included Studies

**Figure 2:**
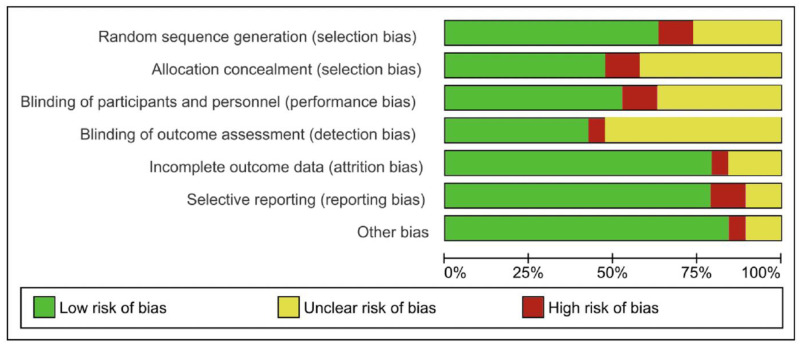
Risk of bias summary: review of the authors’ judgement on the risk of bias for the analyzed randomized controlled trials.

The methodological quality of the 19 included randomized studies was analyzed using Risk of Bias Manager 2.0 included in the RevMan online data tool and is presented next to every forest plot graphically and summarized in Figure 2.

Twelve studies clearly outlined the randomization method, and all were assessed as having a “low risk” for selection bias. Five studies mentioned randomization but did not specify the methods, resulting in “unclear” evaluations for selection bias. Nine studies reported on allocation concealment, with all deemed to have a “low risk” of selection bias, whereas the remaining studies were categorized as “unclear” or “high risk” for this aspect. Ten studies implemented blinding for both investigators and participants, which resulted in a “low risk” for implementation bias, while the other studies were rated as having “unclear” or “high risk” implementation bias. Eight studies used blinding for outcome assessors, leading to a “low risk” of measurement bias, but the others had “unclear” or “high risk” ratings for this type of bias. Fifteen studies addressed missing data bias, reporting bias, and other potential bias sources as having a “low risk”, whereas the other ones had “unclear” or “high risk” ratings for this type of bias.

### 3.4. Impact of Probiotics on Postoperative Liver Function

**Figure 3:**
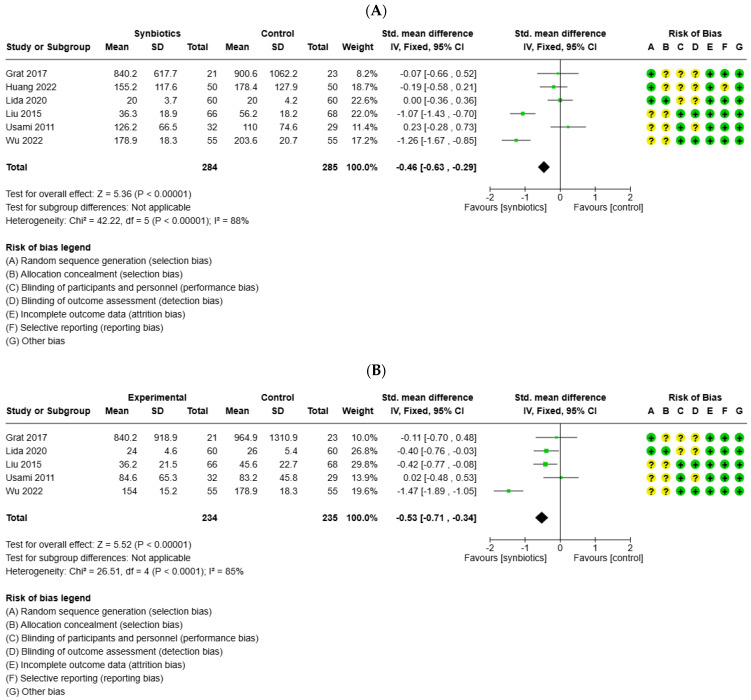

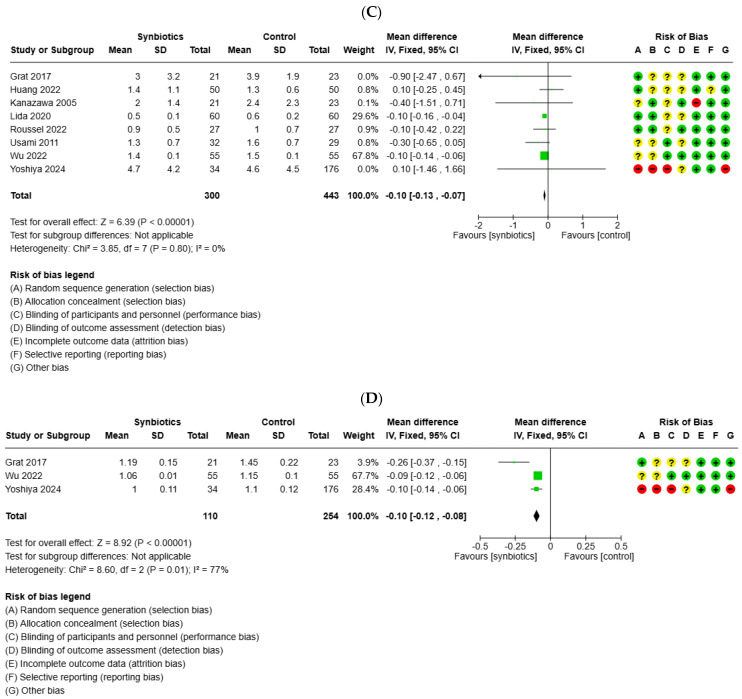
Forest plot for the meta-analysis of postoperative liver function: (**A**) alanine aminotransferase (ALT), (**B**) aspartate aminotransferase (AST), (**C**) total bilirubin, and (**D**) international normalized ratio (INR), (+) low risk of bias, (?) unclear risk of bias and (−) high risk of biasl.

Six studies reported on the levels of ALT, and five studies reported on the levels of AST following liver surgery. The meta-analysis showed significant differences in ALT and AST levels with a favor of the symbiotic group compared to the control group (SMD = −0.46; 95%CI −0.63 to −0.29; *p* < 0.0001) (Figure 3A) (SMD = −0.53; 95%CI −0.71 to −0.34; *p* < 0.0001) (Figure 3B). Eight studies evaluated postoperative bilirubin levels, and three studies analyzed postoperative INR levels. The meta-analysis also indicated significant differences in postoperative bilirubin levels and INR levels with a favorable effect in the synbiotics group compared to the control group (SMD = −0.35; 95%CI −0.50 to −0.19; *p* < 0.0001) (Figure 3C) (SMD = −0.1; 95%CI −0.12 to −0.08; *p* ≤ 0.0001) (Figure 3D).

### 3.5. Impact of Probiotics on Postoperative Inflammatory Markers

**Figure 4:**
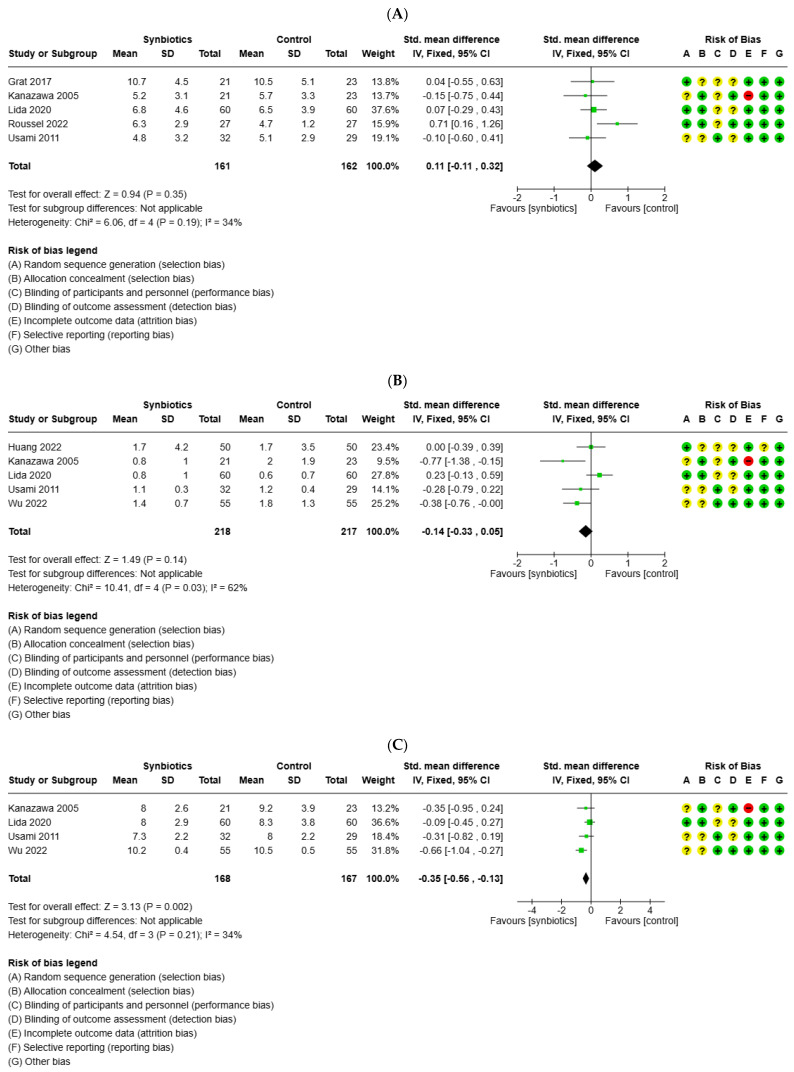

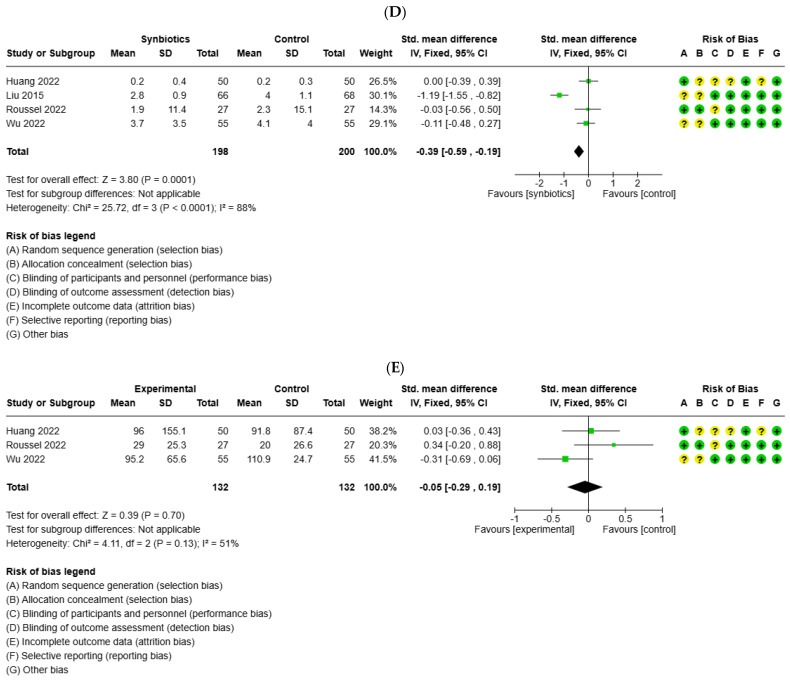
Forest plot for the meta-analysis of postoperative inflammatory indexes: (**A**) C-reactive protein (CRP); (**B**) procalcitonin (PCT); (**C**) white blood cell counts (WBC); (**D**) endotoxin; (**E**) interleukin-6, (+) low risk of bias, (?) unclear risk of bias and (−) high risk of bias.

Five studies examined postoperative CRP levels, five evaluated postoperative PCT levels, and two studies evaluated postoperative IL-6 levels. The meta-analysis consistently found no significant differences in the postoperative levels of these inflammatory markers between the probiotics and control groups (SMD = 0.11; 95%CI −11 to 0.32; *p* = 0.19) (Figure 4A) (SMD = −0.14; 95%CI −0.33 to 0.05; *p* = 0.14) (Figure 4B) (SMD = −0.05; 95%CI −0.29 to 0.19; *p* = 0.70) (Figure 4E).

Four studies reported on white blood cell counts, and a random-effects meta-analysis (I^2^ = 34%) indicated that white blood cell levels were significantly lower in the probiotics group compared to the control group (SMD = −0.35; 95%CI −0.56 to −0.13; *p* = 0.002) (Figure 4C). In addition, four studies reported on endotoxin levels, and a random-effects meta-analysis (I^2^ = 88%) indicated that endotoxin levels were significantly lower in the probiotics group compared to the control group (SMD = −0.39%CI −0.59 to −19; *p* < 0.0001) (Figure 4D).

### 3.6. Probiotic Effects on Postoperative Outcome

**Figure 5:**
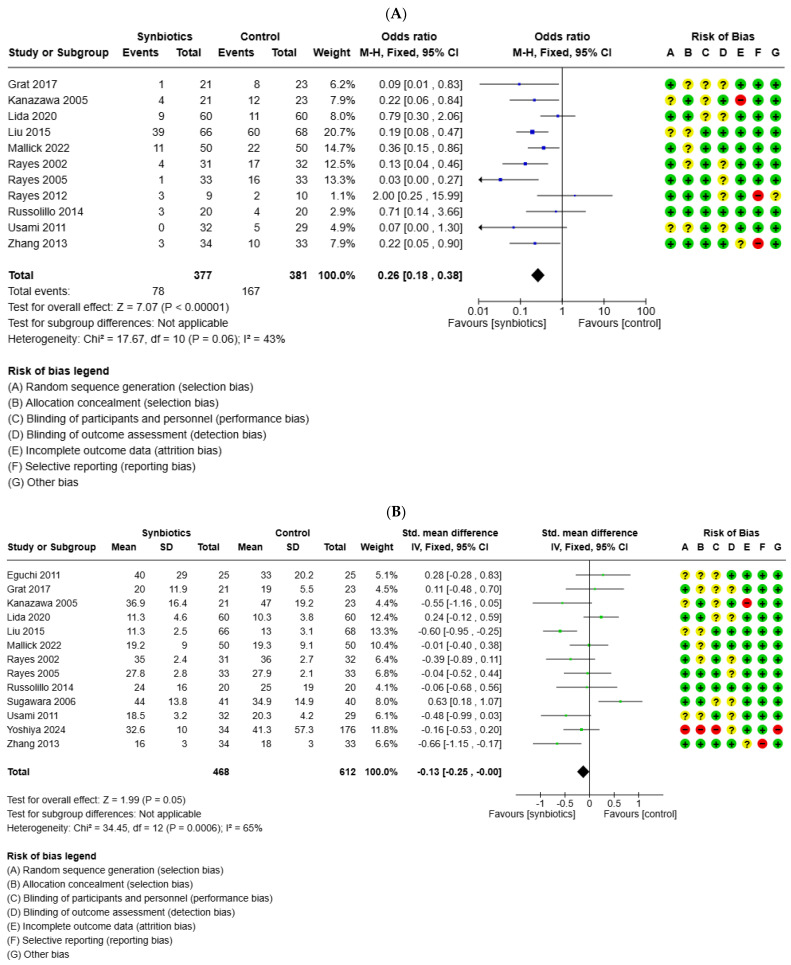

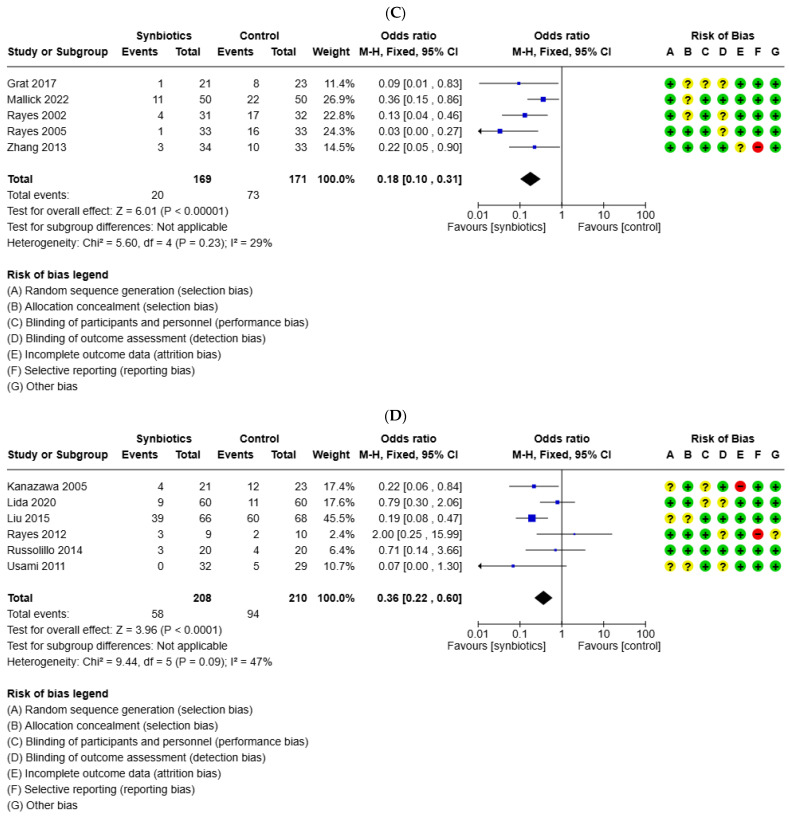
Forest plot for the meta-analysis of postoperative outcome: (**A**) infection complications in all studies, liver transplantation (**C**), and liver resection (**D**); (**B**) length of hospital stay, (+) low risk of bias, (?) unclear risk of bias and (−) high risk of bias.

Fifteen studies documented postoperative complications/infections, with 129 infections out of 530 patients in the probiotics group and 240 out of 533 in the control group. Given low heterogeneity (I^2^ = 44%), a fixed-effect model was applied. The meta-analysis revealed that the incidence of postoperative infections was significantly lower in the probiotics group compared to the control group (OR = 0.34; 95%CI 0.25 to 0.45; *p* < 0.0001) (Figure 5A). The length of hospital stay was reported in thirteen studies. The meta-analysis revealed that the probiotics group had a significantly shorter hospital stay compared to the control group (OR = −0.13; 95%CI −0.25 to −0.00; *p* = 0.05) (Figure 5B). A subgroup analysis in infections, which differentiated between studies including patients with liver transplantation and studies with patients receiving liver resection, was also applied (Figure 5C,D). Results confirmed the initial analysis in both groups.

### 3.7. Sensitivity Analysis

A sensitivity analysis was conducted by excluding each study one at a time to assess its impact on the pooled results. The sensitivity analysis showed that the overall effect size did not change significantly after excluding any individual study, suggesting that the results of the meta-analysis are robust.

## 4. Discussion

This systematic review analyzed 19 randomized controlled trials to evaluate the effects of pro-/synbiotics on postoperative outcomes following liver surgery. The results demonstrated that the perioperative administration of specific microecological regulators (“synbiotics and/or probiotics”) significantly reduces the incidence of postoperative infectious complications and duration of hospital stay in liver surgery patients. Additionally, the study also found out that probiotics effectively lower white blood cell counts and endotoxin levels after surgery, helping to reduce the postoperative inflammatory response. However, no significant differences were observed in the postoperative levels of CRP, PCT, and IL-6. Simultaneously, this study could reveal significant differences in postoperative AST, ALT, bilirubin, and INR indices between the two patient groups. This suggests that the perioperative administration of probiotics may promote the recovery of liver function in the analyzed patient collective.

Previous studies have shown that the disruption of microbiota, caused by surgical stress responses, triggers the release of inflammatory cytokines and increases the permeability of the intestinal barrier. This leads to bacterial translocation and further microbiota imbalance, impairing the liver’s detoxification capacity as a key organ in the gut–liver axis [2,46,47].

Moreover, significant intraoperative blood loss and postoperative liver function impairment increase infection susceptibility, contributing to a higher risk of infections and poor outcomes [4,9]. Postoperative infections are a major factor driving elevated morbidity and mortality rates after hepatectomy and contribute significantly to rising healthcare costs. In fact, up to 30% of patients that undergo hepatectomy experience infectious complications [2,6,9].

After surgery, systemic endotoxinemia is promoted by several factors, including the presence of both aerobic and anaerobic bacteria throughout the gastrointestinal tract, microvascular damage, and weakened gut integrity caused by ischemia-reperfusion injury. Additionally, immune dysfunction due to the inflammatory response, prolonged visceral ischemia (as the intestinal mucosa is more vulnerable to necrosis than other tissues), and the effects of hemorrhage and low blood pressure further contribute to this process. Under these conditions, transient endotoxinemia is almost inevitable, potentially leading to both infectious and non-infectious postoperative complications [2]. Despite the use of perioperative antibiotics, the incidence of postoperative infections remains high, and the effective management of infections upon their occurrence during the postoperative recovery period is crucial for improving patient outcomes after liver surgery [11].

Therefore, identifying and implementing effective interventions to reduce postoperative infections is of considerable clinical importance. The primary roles of probiotics, prebiotics, and synbiotics include enhancing colonization resistance against harmful microbes, improving bowel motility and blood circulation in the splanchnic region, promoting enterocyte growth and mucus production, regulating intestinal inflammation, strengthening the gut barrier, and supporting both immune and non-immune defense mechanisms by competing with potential pathogens [41]. Probiotics influence intestinal immune function by reducing pro-inflammatory cytokines, promoting tolerance-inducing cytokine profiles and regulatory pathways, and increasing secretory IgA levels. They also support epithelial cell homeostasis by strengthening the gut barrier, enhancing cytoprotective responses, improving cell survival, and boosting mucin production. Pathogenic microbes are counteracted through multiple mechanisms, including reducing their adhesion to the mucosa, lowering luminal pH, and producing antibacterial bacteriocins. Probiotics also contribute to nutrition by aiding in the breakdown of indigestible food components and enhancing nutrient absorption [2,3].

Additionally, they play a role in neuromodulation by activating cannabinoid and opioid receptors on epithelial cells, a mechanism observed in *Lactobacillus acidophilus*, though current evidence is based on animal studies, with human data still lacking [48]. Beyond this, probiotics help reduce visceral hypersensitivity and regulate stress responses, including stress linked to surgical procedures, known as the “surgical stress reaction” [49].

A previous review by Moran et al. found no substantial benefits of synbiotics in elective abdominal surgery. However, in patients undergoing hepatopancreatobiliary procedures or liver transplantation, synbiotic use was associated with a significant reduction in postoperative infectious complications [50]. However, with a focus on liver surgical procedures, the stage of malnutrition is a common risk factor for postoperative complications and infections in patients with liver cirrhosis undergoing liver resection or transplantation. The gut–liver interaction is well established, with intestinal dysbiosis and increased gut permeability contributing to microbial overgrowth. This process allows bacteria, fungi, and their byproducts—such as endotoxins from gram-negative bacteria and beta-glucans from fungi—to translocate into the portal venous system. When the intestinal barrier is compromised under pathological conditions, the increased translocation of microbial products triggers an inflammatory response. This inflammation can cause liver damage, impair hepatocyte function, and reduce the liver’s detoxification capacity, potentially contributing to the development of inflammatory liver diseases [2].

In a recent research study conducted by Xia et al., oral probiotics significantly reduced serum ammonia and endotoxin levels in patients with liver cirrhosis and helped prevent the overgrowth of *Escherichia coli* and *Staphylococcus* in the intestines [51,52]. Similarly, Wibawa et al. found that oral microbiotics could alleviate mild hepatic encephalopathy and improve liver function [53]. Further, fermentable fibers were also effective in some patients following liver surgery for colorectal liver metastases [43]. Our findings suggest that perioperative probiotic/symbiotic administration may reduce the incidence of postoperative infections, decrease white blood cell counts, and lower serum endotoxin levels.

AST and ALT, markers of hepatocellular injury, usually peak during the early postoperative phase. Bilirubin levels are predictive of liver dysfunction after surgery and are critical indicators of perioperative outcomes [54,55]. Findings of our meta-analysis indicate that the administration of these microregulatory agents can significantly support postoperative liver function restoration (ALT; AST; bilirubin: ↓; INR↑) and reduce hospital stay duration. Previous research has shown that infections are a significant factor in determining the length of hospitalization [1,9]. Probiotic use may reduce infections post-hepatectomy, which could explain the shortened hospital stay observed in our study.

Infections are even more common in patients with liver cirrhosis and/or after liver transplantation due to immunosuppression [2,56,57]. In liver transplantation, factors such as malnutrition, ischemia-reperfusion injury, and immunosuppressive therapy can contribute to dysbiosis, a weakened intestinal barrier, impaired innate immune response, and bacterial translocation. These disruptions may increase the risk of early infections, graft failure, and reduced survival. The shift in gut microbiota, characterized by a loss of beneficial bacteria and an overgrowth of harmful species, leads to elevated endotoxin levels and heightened bacterial translocation, further exacerbating these complications. Previous research indicates that the intestinal microbiota influence liver tumor development and inflammatory responses by modifying the activity of pro-inflammatory microorganism-associated molecular patterns, bacterial metabolites, and natural killer (NK) T cell-mediated bile acid metabolism. Additionally, the microbiota contribute to the suppression of antitumor immunity through prostaglandin (PG)E2-mediated mechanisms [58,59]. After liver surgical approaches, macrophages and monocytes release inflammatory mediators in response to stress [60]. IL-6, a key regulator of inflammation, stimulates the release of B and T lymphocytes [61]. High IL-6 levels promote the production of CRP in the liver, which is linked to infection, trauma, and cardiovascular and renal complications [62,63,64].

Additionally, we did not observe a significant reduction in CRP, procalcitonin, IL-6, or other inflammatory markers following probiotic administration. This lack of effect may be attributed to the primary role of probiotics, which is to support immune function and metabolic balance by modulating gut microbiota rather than directly suppressing systemic inflammation [64,65]. Moreover, the systemic inflammatory response in surgical patients is influenced by a variety of complex factors that go beyond the scope of probiotic action. These include the extensive physiological stress induced by surgical trauma, anesthesia-related immune modulation, significant blood loss leading to compensatory inflammatory reactions, and ischemia-reperfusion injury, which triggers a cascade of pro-inflammatory mediators. Additionally, perioperative factors such as fluid management, antibiotic use, and pre-existing conditions may further contribute to inflammatory responses, potentially overshadowing any anti-inflammatory effects of probiotics. Given these multiple interacting influences, it is likely that probiotics alone are insufficient to induce a measurable reduction in inflammatory markers in this setting [2,60,66,67,68].

However, there are several limitations in this study. First, despite including 19 studies, the overall sample size is relatively small, which may affect the reliability of the conclusions. Second, while all the included studies were RCTs, some did not provide adequate information on allocation concealment or blinding procedures, reducing the methodological rigor. Third, although all interventions involved oral probiotics/synbiotics, the duration of treatment and dose of given medications varied across studies, potentially influencing postoperative recovery and introducing clinical heterogeneity. Due to the high heterogeneity of the data with unclear and non-reproducible parameters, it is unfortunately not possible to conduct a conclusive and adequate analysis and make valid statements. Fourth, when analyzing certain indicators, such as CRP, white blood cell count, procalcitonin, IL-6, endotoxin levels, and other inflammatory markers, a limited number of studies were available, leading to high heterogeneity. Therefore, the meta-analysis results for these indicators should be interpreted with caution, and further research is suggested to explore and investigate effects across these study aspects.

## 5. Conclusions

The findings of this meta-analysis suggest that perioperative probiotic/synbiotic administration may lower the risk of postoperative infections and reduce hospital stay duration for patients that undergo hepatectomy. In addition, we could further show that pro-/synbiotics support postoperative liver function recovery and decrease inflammatory markers. However, the existing evidence on the preventive use of probiotics or synbiotics during the perioperative period in liver surgery remains inconsistent. This is due to the lack of standardized formulations, variations in the duration of administration, differences in delivery methods, and the absence of uniform study controls, such as standard care or placebo. These inconsistencies make it difficult to compare findings across different studies.

Although perioperative probiotics show potential benefits, additional high-quality research is required to fully understand their mechanisms and effects in detail.

## Data Availability

The data analyzed during this study can be found within the published article. All data from this study are available upon reasonable request to the corresponding author.
